# Immunological Hallmarks for Clinical Response to BCG in Bladder Cancer

**DOI:** 10.3389/fimmu.2020.615091

**Published:** 2021-01-29

**Authors:** Chun Jye Lim, Phuong Hoang Diem Nguyen, Martin Wasser, Pavanish Kumar, Yun Hua Lee, Nurul Jannah Mohamed Nasir, Camillus Chua, Liyun Lai, Sharifah Nur Hazirah, Josh Jie Hua Loh, Li Yan Khor, Joe Yeong, Tony Kiat Hon Lim, Alvin Wei Xiang Low, Salvatore Albani, Tsung Wen Chong, Valerie Chew

**Affiliations:** ^1^ Translational Immunology Institute (TII), SingHealth-DukeNUS Academic Medical Centre, Singapore, Singapore; ^2^ Duke-NUS Medical School, Singapore, Singapore; ^3^ Division of Pathology, Singapore General Hospital, Singapore, Singapore; ^4^ Institute of Molecular Cell Biology (IMCB), Agency of Science, Technology and Research (ASTAR), Singapore, Singapore; ^5^ Department of Urology, Singapore General Hospital, Singapore, Singapore

**Keywords:** Bacillus Calmette–Guerin (BCG), bladder cancer, Immunotherapy, biomarkers, PD-1

## Abstract

Intravesical Bacillus Calmette-Guerin (BCG) is an effective immunotherapy for non-muscle invasive bladder cancer (NMIBC). However, recurrence and progression remain frequent warranting deeper insights into its mechanism. We herein comprehensively profiled blood and tissues obtained from NMIBC patients before, during and after BCG treatment using cytometry by time-of-flight (CyTOF) and RNA sequencing to identify the key immune subsets crucial for anti-tumor activity. We observed the temporal changes of peripheral immune subsets including NKT cells, central memory CD4^+^ T cells, CD8^+^ T cells and regulatory T cells (Treg) during the course of BCG. Gene expression analysis revealed enriched immune pathways involving in T cell activation and chemotaxis, as well as a more diversified T cell receptor repertoire in post-BCG tissues. Moreover, tissue multiplexed-immunofluorescence (mIF) showed baseline densities of non-Treg and CD8^+^PD-1^+^ T cells were predictive of response and better recurrence-free survival after BCG. Remarkably, post-BCG tissues from responders were found to be infiltrated with more active CD8^+^PD-1^-^ T cells and non-Treg CD4^+^FOXP3^-^ T cells; but increased exhausted CD8^+^PD-1^+^ T cells were found in non-responders. Taken together, we identified predictive biomarkers for response and uncovered the post-treatment expansion of exhausted PD-1^+^CD8^+^ T cells as key to BCG resistance, which could potentially be restored by combining with anti-PD-1 immunotherapy.

## Introduction

Urothelial carcinoma (UC) is the major type of bladder cancer, which can be subdivided into non-muscle invasive (NMIBC) or muscle invasive (MIBC) bladder cancer. At diagnosis, 75% of patients have NMIBC and 25% have MIBC (1). The majority of disease-related mortality is due to the more aggressive MIBC, in which the tumors invade the detrusor muscle (2). Depending on the stage and grade of the NMIBC, adjuvant therapy such as Bacillus Calmette-Guerin (BCG) immunotherapy is recommended as a strategy to prevent recurrence and reduce risk of progression to MIBC after transurethral resection of bladder tumor (TURBT) (3). BCG therapy, which involves instilling an attenuated strain of Mycobacterium bovis intravesically to the bladder a few weeks after TURBT, was one of the earliest forms of cancer immunotherapies (4). Despite treatment, 30-50% of patients receiving BCG fail to respond and 10-15% experience progression to MIBC (5). Therefore, predicting high-risk patients who might not benefit from BCG treatment is critical. Failure to detect recurrence and the resulting delay of radical surgery for these patients could worsen their survival outcome (6).

Better understanding of the mechanism of BCG treatment related to response would be critical for novel therapeutic design. Thus far, both CD4 and CD8 T cells have been shown critical for BCG-mediated anti-tumor activity and response in mouse models (7, 8). In humans, the role of TILs, particularly the predisposition of Th2 CD4 T cells in tumor have been implicated in response to BCG treatment (9–11). There were also reports showing that the post-BCG tissues were infiltrated with increased numbers of both CD4^+^ and CD8^+^ T cells (12–14). Despite widespread infiltration of these cells, tumor recurrence after BCG failure remains frequent. This warrants a deeper interrogation of the underlying immune and transcriptomic landscape associated with BCG treatment, which may provide clues on the mechanism of BCG as well as the identification of patients who may best benefit from this immunotherapy.

In this study, we aim to study the immunome changes in peripheral blood which we believe could reflect the immune response in the tumor microenvironment induced by BCG treatment. By employing the high-dimensional single cell analysis *via* cytometry by time-of-flight (CyTOF), we examined the temporal changes of immune cells in peripheral blood before and at different time points after BCG treatment. Indeed, we discovered the changes in both frequencies and phenotypes of various immune subsets such as CD4 and CD8 T cells in the peripheral blood after BCG treatment, indicating a systemic immune response. Transcriptome analysis of pre and post-BCG tissues revealed gene signature involved in T cell activation and recruitment as well as increased diversity of TCR repertoire in urothelial microenvironment following BCG treatment. Our analysis from multiplexed immunofluorescence (mIF) data from an independent cohort revealed that densities of baseline CD4^+^FOXP3^-^ non-Treg cells and CD8^+^PD-1^+^ were higher in responders and predicts for better recurrence free survival (RFS) in NMIBC patients after BCG treatment. Finally, we validated that post-BCG tissues were presented with higher densities of CD4^+^FOXP3^-^ non-Treg cells and PD-1^-^ non-exhausted or active CD8^+^ T cells in responders; whereas BCG-induced expansion of PD1^+^CD8^+^ T cells was linked to non-responsiveness to therapy.

## Materials and Methods

### Study Approval and Specimens

Five patients with NMIBC who underwent transurethral resection to remove all endoscopically visible tumors followed by BCG instillations in Singapore General Hospital (SGH) were recruited upon informed consent according to guidelines from institutional review board (IRB). The patients’ baseline clinicopathological parameters were analyzed (**Supplementary Table S1**). After two-four weeks, the patients received weekly intravesical BCG (12.5mg of Tice BCG strains) instillations for six times as standard protocol (3). Upon completion of the 6 doses instillations, surgical specimens were obtained to confirm that no neoplastic pathology of the mucosa by the pathologists. Tissue specimens were obtained from: pre-BCG resected tumor (T), pre-BCG adjacent non-tumor tissue (NT) and post-BCG NT urothelial tissue. Blood specimens were obtained before (pre) and two time points after BCG instillations at 1 month (1M) and 3 months (3M). Two patients (BCGx09 and BCGx15) withdrew from the study and stopped BCG treatment before the last time point due to BCG toxicities/intolerance.

A small piece of pre- and post-BCG tissues were subjected to RNA sequencing analysis. Tissue-infiltrating leukocytes (TILs) were isolated from pre- and post-BCG tissues with enzymatic digestion: 100μg/ml Collagenase IV (ThermoFisher Scientific, USA) and 100μg/ml DNase1 (Sigma-Aldrich) and peripheral blood mononuclear cells (PBMCs) from blood using Ficoll-Paque layering, both as previously described (15). TILs were analyzed using flow cytometry and PBMCs were stored with 10% DMSO in liquid nitrogen until later analysis with CyTOF.

From another independent cohort of 29 NMIBC patients, a total of 45 archival formalin-fixed, paraffin-embedded (FFPE) tissue specimens before (n= 29) and after (n=16) BCG induction, were obtained and analyzed from the Department of Anatomical Pathology of SGH. Their clinicopathological parameters were reviewed (**Supplementary Table S2**).

For both cohorts, we defined responders as patients who did not show recurrence: demonstrated an absence of disease on cytology, cystoscopy, or random biopsies at 24 months since the last BCG exposure. As per IBCG (International Bladder Cancer Group (16) guidelines, we defined BCG maintenance as at least two or three courses of BCG at first maintenance.

### Cytometry by Time-of-Flight Staining and Analysis

PBMCs were stained with 37 metal-conjugated antibodies (Lymphoid panel, **Supplementary Table S3**) as previously described (15, 17). After staining, data acquisition was done on the Helios CyTOF mass cytometer (Fluidigm). The generated files were analyzed using FlowJo v10.5.1 (FlowJo, USA) and down-sampled to equal number of live immune cell events of 12906 (smallest possible events of the 13 files) for comparison. To identify cell populations, we clustered the merged data containing 167778 single cell events using the FlowSOM (18) method. Clusters showing significant temporal frequency changes were detected using repeated measures ANOVA, where linear mixed models were used to account for missing data (19). Clusters were assigned to 7 major cell lineages and visualized with t-stochastic neighbor embedding (t-SNE) (20). Clustering results were validated by manual gating using FlowJo v10.5.1. Statistical analysis and data visualization were performed with R packages (21). In another CyTOF experiment to investigate MDSC, PBMC were stained with metal-conjugated antibodies (Myeloid panel, **Supplementary Table S3**) as described above. The CyTOF data were manually gated to check for the frequencies of MDSC using FlowJo.

### Next-Generation Sequencing

Tissue samples (n=8) from three pairs matched of pre- and post-BCG samples and another two unmatched pre-BCG samples, were stored in RNAlater (Thermo Fisher Scientific, USA) at -80°C until further processing. RNA was isolated using the mirVana miRNA Isolation kit (Thermo Fisher Scientific, USA) and cDNA was generated with the SMART-Seq v4 Ultra Low Input RNA kit for Sequencing (Clontech, USA), according to manufacturer’s instructions. Illumina-ready libraries were generated using the Nextera XT DNA Library Prep Kit (Illumina, USA), multiplexed for 2x 101 bp-sequencing and sequenced on a HiSeq High-throughput platform at the Genome Institute of Singapore. Raw reads obtained from sequencing were mapped *via* Hierarchical Indexing for Spliced Alignment of Transcripts taking Genome Reference Consortium Human Build 38 patch release 7 as a reference. Reads were then sorted with SAMtools and high-throughput sequencing data was used to obtain the gene counts (22) which were then analyzed for differential gene expression (DEG) between the matched pre and 3M post BCG groups using the R package EdgeR tool. Paired analysis was done by using the empirical Bayes quasi-likelihood F-test in the Generalized Linear Model pipeline for testing of differential expressed genes (DEGs, p-value < 0.05) on samples with available matched pre- and post-BCG tissues (23). Analysis of biological function was performed using the Database for Annotation, Visualization and Integrated Discovery (DAVID) V.6.8 Functional Annotation Tool. Only non-redundant biological processes with a Benjamini-Hochberg adjusted p-value < 0.05 were reported. Sequence data used in the study has been deposited at the European Genome–phenome Archive (EGA) under the accession codes: EGAS00001004764.

### TCR Repertoire Analysis

Raw reads from RNA-sequencing were aligned and sequences of TCR clones sequence were extracted using the MiXCR software (24). Clonal homeostasis, top clonal proportions, Shannon index and Chao1 index were performed using methods from tcR R package (25). TCR raw reads from one pre-BCG tumor tissue of a patient were extremely low and not reliable, and hence, were excluded from analysis. A total of seven samples: two pairs of matched pre and post-BCG samples, two unmatched pre-BCG and one-unmatched post-BCG sample were included in analysis. Unpaired Student’s t-test was performed on the remaining dataset.

### Flow Cytometry

Due to the limited size of the fresh tissues, immune cells isolated from tumor or non-tumor tissues were analyzed with flow cytometry instead of CyTOF. The cells were rested for 30mins at 37°C and then labeled with antibodies against surface markers: CD45, CD3, CD4, CD8, CD56, CD16, gdTCR, CD27, PD-1, and CD69 (see **Supplementary Table 4** for antibody panel). Stained samples were then analyzed using LSRFortessa™ flow cytometer (BD Bioscience) and data analysis was performed using FlowJo v10.5.1.

### Multiplexed Tissue Immunofluorescence

Multiplexed IF was performed on a total of 45 FFPE samples (29 pre-BCG tumor and 16 post-BCG specimens) with the Opal system and images were acquired using the Vectra 3.0 Automated Quantitative Pathology Imaging System (Perkin Elmer) with 4′,6-diamidino-2-phenylindole (DAPI) as the nuclear marker as previously described (26). The antibodies used are anti-Human CD4 (clone EPR6855; Abcam), anti-Human CD8 (clone C8/144B; DAKO), anti-Human FOXP3 (clone 236A/E7; Abcam), and anti-Human PD-1 (clone NAT105; Abcam). From these 45 FFPE samples, another single-plexed staining of PD-L1, anti-human PD-L1 antibody (clone 22C3; DAKO), was separately performed on consecutive slide of 12 samples (six pairs of matched pre- and post-BCG tumor from non-responders). Full tissue sections were used for all samples. For specimens smaller than 1cmx0.5cm, whole tissue was imaged and quantified; whereas for specimens larger than 1cmx0.5cm, at least 10 areas of 2 mm x 3 mm with high infiltration of immune cells were imaged and quantified. Quantification was done by ImageJ and the mean was calculated as the cell density (number/mm^2^) for each patient sample, while area with PD-L1 positive staining was quantified and the mean was reported as PD-L1+area/mm^2^. The median value of density for each immune subsets was used as the cutoff point to dichotomize the patients into two groups (low versus high).

### Statistics

For FlowSOM Clusters, the significant changes were detected using repeated measures ANOVA (19). Univariate analysis was performed using the cox proportional hazards model. All the statistical analyses listed below were performed in GraphPad Prism v7.0d. For manually gated CyTOF data with the matched time points (pre, 1M and 3M for BCGx02, BCGx10, BCGx16), non-parametric One-way ANOVA by Friedman’s test followed by Dunn’s post-test was used. RNA sequencing data from eight tissue samples were analyzed for *CXCL9* gene expression and TCR clonality analysis by Mann-Whitney U test. For mIF tissue data analysis, two-tailed Wilcoxon matched-pairs signed rank test was used for matched pre vs post tissues while Mann-Whitney U test was used for responders versus non-responders. Spearman correlation was used for correlation of PD-L1 expression and CD8+PD-1+ density in pre- and post-BCG tumor tissues from non-responders. Kaplan Meier analysis for recurrence free survival (RFS) was performed with the log-rank (Mantel-Cox) test.

## Results

### Single-Cell Mass Cytometry Analysis on Temporal Changes of Peripheral Immune Subsets Upon Bacillus Calmette–Guerin Treatment

**Figure 1:**
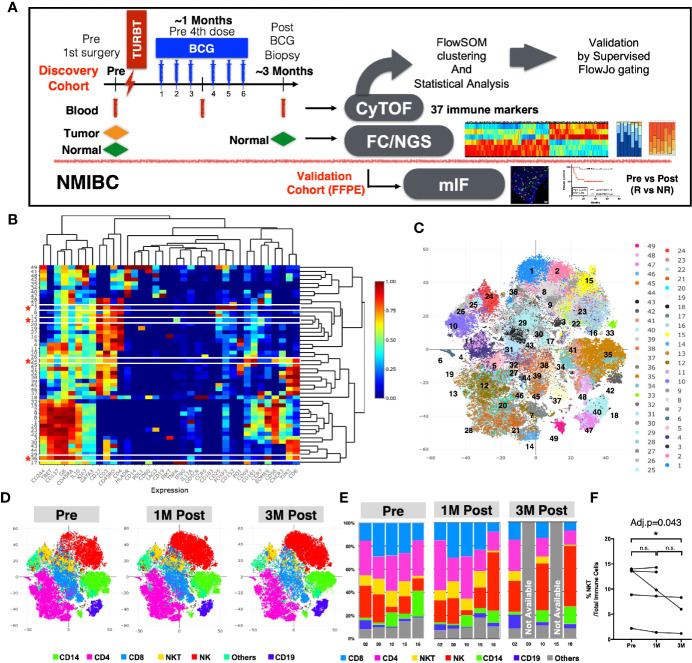
Immune profiles of peripheral blood mononuclear cells (PBMCs) before and after BCG treatment. **(A)** Schematic diagram of the experimental design and analysis workflow. **(B)** Heatmap depiction of 49 FlowSOM clusters with normalized protein expression (arcsine scaled) of all 37 markers from 13 PBMC samples. *denotes the four clusters significantly different among three time points by repeated measure ANOVA statistical test. **(C)** Visualization of 49 FlowSOM clusters in a t-SNE map, each clusters represented by different colors. **(D)** t-SNE maps showing major immune lineages, each indicated by one color, at Pre, 1M Post-BCG and 3M Post-BCG time points. **(E)** Bar charts for manually gated proportions of major immune lineages from PBMCs of five patients at Pre, 1M and 3M Post-BCG time points. **(F)** Line plot showing the frequencies of NKT cells over total immune cells at three time points from all patients. Connected line specifies samples from same patient at different time points. *adjusted p<0.05 with non-parametric One-way ANOVA by Friedman’s test followed by multiple pairwise comparison using Dunn’s test for matched data points with complete set of three time points. BCG, Bacillus Calmette-Guerin; TURBT, transurethral resection of bladder tumor; NMIBC, non muscle-invasive bladder carcinoma; NGS, next generation sequencing; FC, flow cytometry; mIF, multiplexed immunofluorescence imaging; R, Responders; NR, non-responders.

We hypothesized that the BCG treatment could induce immunome changes in the tumor microenvironment that would be reflected in peripheral blood. In order to screen the systemic changes of the immune landscape induced by intravesical immunotherapy with BCG in NMIBC patients, we performed an in-depth high-dimensional single cell analysis *via* CyTOF on PBMC collected from NMIBC patients (Clinical info summarized in **Supplementary Table S1**) across three time-points: before undergoing Transurethral Resection of the bladder tumor (TURBT) and receiving BCG (Pre), at 1M post-BCG after receiving three doses of BCG (1M Post), and at 3M upon completion of six doses of BCG (3M Post) (**Figure 1A**). The PBMCs were stained with an antibody panel of 37 leukocyte specific markers covering major immune lineages and markers related to immune cell functions (**Supplementary Table S3**). After selecting live cells and sample debarcoding as previously described (15), we merged the CyTOF data (five patients in three time points, see Methods) and performed clustering using the FlowSOM algorithm (18). Heatmaps reveal the differential expression of all 37 markers in 49 clusters (**Figure 1B**), which were also visualized by t-stochastic neighbor embedding (t-SNE) map (20) (**Figure 1C**). The same t-SNE map, was also used to show the relative expression of all 37 markers individually (**Supplementary Figure S1**).

We first annotated the clusters based on the differential expression of six lineage markers (CD3, CD4, CD8, CD56, CD19, and CD14) to define the six major immune lineages: CD4^+^ T cells, CD8^+^ T cells, CD56^+^ NK cells, CD3^+^CD56^+^ NKT cells, CD19^+^ B cells, CD14^+^ cells and others. The abundance of each immune lineage at each time point is shown by the t-SNE maps (**Figure 1D**). To validate this result, we also manually gated out all immune lineages using FlowJo (**Figure 1E** and **Supplementary Figure S2A**). We observed significant decrease in frequency of NKT cells comparing PBMCs from pre to 3M post-BCG (**Figure 1F**). Other immune lineages including HLA-DR+ antigen presenting cells did not appear to show any difference (**Supplementary Figure S2B**). As myeloid-derived suppressor cells (MDSC) has been implicated in bladder cancer (27), we then performed an additional MDSC profiling using CyTOF. However, both polymorphonuclear- and monocytic-MDSC (PMN-MDSC and M-MDSC) showed no signficant difference in frequencies after BCG treatment, despite a trend of post-treatment downregulation (**Supplementary Figures S2C, D**).

### Bacillus Calmette–Guerin Treatment Alters the Frequencies of T and NKT Subsets in Peripheral Blood

**Figure 2:**
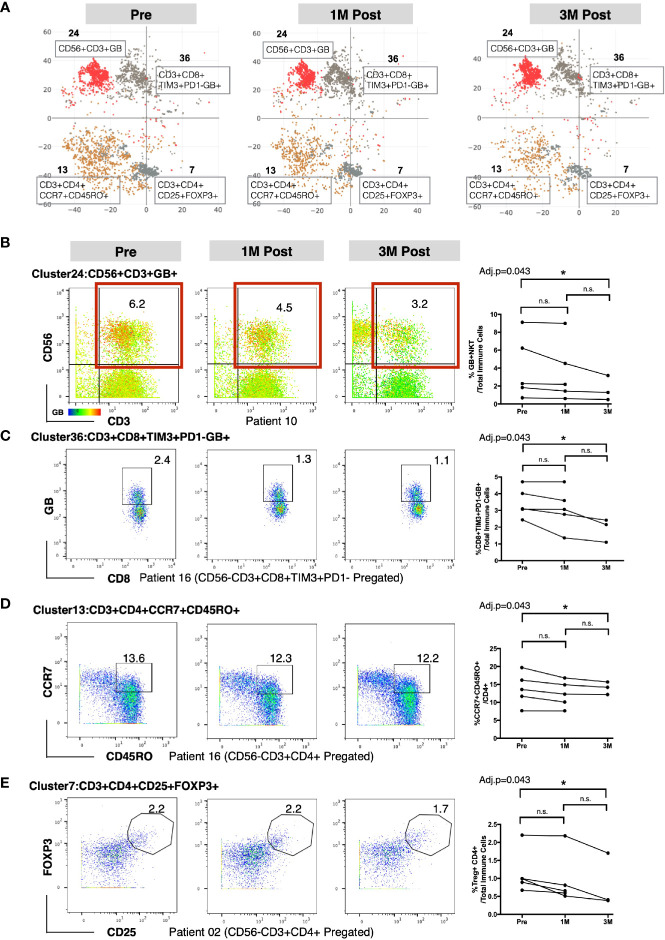
Altered frequencies in immune subsets in peripheral blood mononuclear cells (PBMCs) after Bacillus Calmette-Guerin (BCG) treatment. **(A)** t-SNE maps showing four clusters significantly different at three time points using repeated measure ANOVA test. **(B)** Left, representative dot plots for NKT cells (red boxed) colored by intensity of Granzyme B (GB) expression (cluster 24) from patient 10. Right, line plot showing the frequencies of GB+NKT cells over total immune cells at three time points from all patients. **(C)** Left, representative dot plots for GB expressing PD-1^-^TIM3^+^CD8^+^ T cells (cluster 36) from patient 16. Right, line plot showing the frequencies of GB^+^PD-1^-^TIM3^+^CD8^+^ T cells over total immune cells at three time points from all patients. **(D)** Left, representative dot plots for central memory CD4^+^ T cells from patient 16. Right, line plot showing the frequencies of CD4^+^CCR7^+^CD45RO^+^ T cells (cluster 13) over CD4^+^ T cells at three time points from all patients. **(E)** Left, representative dot plots for Treg cells (cluster 7) from patient 02. Right, line plot showing the frequencies of Treg cells over total immune cells at three time points from all patients. B–E, connected line specifies samples from same patient at different time points. *adjusted p<0.05 or not significant (n.s.) by non-parametric One-way ANOVA by Friedman’s test followed by Dunn’s post-test for matched data points with complete set of three time points.

To identify the specific immune subsets that showed significant temporal changes among the 49 immune clusters, we applied repeated measures analysis of variation (ANOVA) to compare the frequencies of each cluster across the three time points. Four immune clusters were identified to be significantly different in frequency among the three time points (**Supplementary Table S5**). Based on the immune markers expression levels by each cluster (marked with red asterisks in **Figure 1B**), we manually annotated these four clusters as: GB-expressing CD3^+^CD56^+^ NKT cells (Cluster 24); GB-expressing PD-1^-^Tim3^+^CD8^+^ T cells (Cluster 36); central memory CD4^+^ T cell expressing CD3^+^CD4^+^ CCR7^+^CD45RO^+^ (Cluster 13); and regulatory CD4^+^ T cell (Treg) expressing CD3^+^CD4^+^CD25^+^FOXP3^+^ (Cluster 7); (**Figure 2A**). In general, we observed a decreasing trend for these clusters post-BCG (**Figure 2A**). To further identify the time point at which the difference in frequency occurred for these four immune subsets, we then performed manual gating with FlowJo and used the multiple pairwise comparison with Dunn’s test on all the available matched data. The frequency of GB^+^ NKT cells was shown to be decreased from pre compared to 3M post-BCG blood (**Figure 2B**). Furthermore, the frequency of GB-expressing PD-1^-^Tim3^+^, potentially active and cytotoxic CD8 T cells were also significantly reduced at peripheral blood at 3M post-BCG compared to Pre-BCG (**Figure 2C** and **Supplementary Figure S3A**). Finally, circulating central memory CD4 T cells were decreased at 3M post-BCG compared to pre-BCG (**Figure 2D** and **Supplementary Figure S3B**). Interestingly, a distinct reduction in frequency of Treg cells was observed at 3M post-BCG as compared to pre-BCG (**Figure 2E**). In summary, here we described four functionally important subsets which have been altered in frequency, mainly reduced, in the peripheral blood of NMIBC patients, which suggest tissue recruitment upon BCG treatment.

### Transcriptomic Signature of Post Bacillus Calmette–Guerin-Treated Tissues Is Indicative of T Cell Activation, Immune Costimulation, and Chemotaxis Pathways

**Figure 3:**
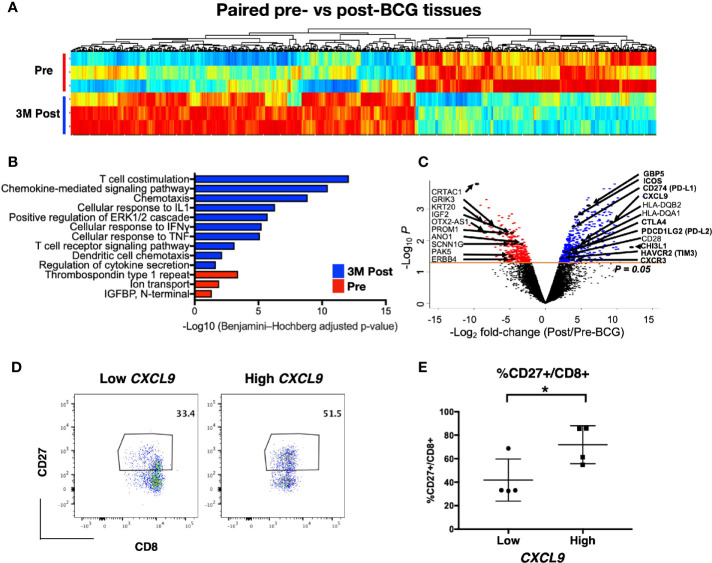
Post-Bacillus Calmette-Guerin (BCG) treated tissues show upregulation in genes involved in multiple immune activation pathways. **(A)** Heatmap showing all differentially expressed genes (DEGs) from matched tissues between Pre and 3M Post-BCG time points (n=3 from each group). **(B)** Functional pathways annotated by DAVID pathway enrichment analysis of the genes enriched in Pre (red bar) or 3M Post-BCG time points (blue bar). **(C)** Volcano plot showing DEGs in the Pre and 3M Post-BCG tissues. Genes enriched in Post-BCG time point are colored in blue while genes enriched in Pre time point are colored in red. Selected genes are highlighted. The orange line denotes the p value of 0.05. **(D)** Representative dot plots showing CD8^+^CD27^+^ cells gated from CD3^+^CD56^-^ T cells of isolated cells from tissues with low (patient 16 pre-NT) and high (patient 2 post-NT) *CXCL9* genes, respectively. Median value for *CXCL9* genes was used as the cutoff point for dichotomisation into two groups. **(E)** Proportion of CD27 expressing CD8 T cells from tissues with low *CXCL9* group (n=4), and high *CXCL9* group (n=4), respectively. Graph shows mean with standard deviation. *p<0.05 by Mann-Whitney U test.

To investigate the transcriptomic changes in the local tissue microenvironment after intravesical BCG instillation, we next performed RNA sequencing on tissues (both tumor and normal) at pre and 3M post-BCG. We examined the differential expressed genes (DEGs) profiles, comparing matched tissues from 3M post-BCG to those from pre-BCG (**Figure 3A**). All DEGs were also subjected to DAVID pathway analysis (**Supplementary Table S6** and **Figure 3B**). Pre-BCG tissues were enriched with genes related to bladder cancer progression, such as *CRTAC1* (28), *ERBB4* (29), *KRT20* (28, 30), *OTX-AS1* (31), *SCNN1G* (30); and ion transport related molecules, such as *ANO1* (32), *GRIK3* (33), which have been shown to associate with poor prognosis in other cancers (**Figures 3B, C**). Conversely, genes enriched in post-BCG tissues were mostly involved in immune response related pathways, indicating that BCG treatment could induce an active immune response (**Figures 3B, C**). Specifically, post-BCG tissues were enriched with the genes involved in chemotaxis and chemokine-mediated signaling pathways such as *CXCL9, CCL18, CCL20, CXCR3*, and *CXCR4* (**Figures 3B, C**), indicative of the recruitment of immune cells such as CD4 and CD8 T cells (34). Besides, T cell costimulation genes like *ICOS* and *CD28* as well as genes involved in T cell inhibitory function such as *CTLA4*, and *HAVCR2(TIM3), CD274(PD-L1), PDCD1LG2 (PD-L2)* were upregulated in post-BCG tissues (**Figure 3C**). Genes that are associated with antigen presentation like *HLA-DQA1* and *HLA-DQB2* were identified to be enriched in post BCG tissues, indicating the activation of T cells (**Figure 3C**). This antigen presentation-related genes together with the T cell costimulation pathway, implied that T cells are one of the critical immune cells possibly recruited to the local tissues after BCG treatment. IFN-γ induced small GTPase families such as *GBP5*, which was previously reported to be upregulated after repeated intravesical BCG treatment in mouse bladder (35) was also among the upregulated genes after BCG treatment (**Figure 3C**). One of the top listed genes by fold change, *CHI3L1* (**Figure 3C**), was described to interact with a carbohydrate polymer (chitin) and involved in the exacerbation of intestinal inflammation by enhancing bacterial/colonic epithelial cells interaction (36). This could be one of the mechanisms for BCG-induced pro-inflammatory microenvironment. Furthermore, pathways like cellular response to IFN-γ and TNF-α also suggested that the local microenvironment is highly pro-inflammation after BCG treatment. Taken together, the current data suggested BCG treatment induced a pro-inflammatory urothelial microenvironment with T cells tissue recruitment and activation. To validate this, we performed flow cytometry on tissues-infiltrating leukocytes from tissues taken at 3M post-BCG and indeed frequencies of CD27 expressing CD8 T cells correlated to the expression level of *CXCL9* gene, a chemokine responsible for attracting CD8+ T cells to inflamed tissue (**Figures 3D, E**). This suggests that our earlier CyTOF data showing the reduction of a number of T cell subsets from the peripheral blood, could correspond to immune cell tissue recruitment after BCG treatment.

### Increased Diversity of TCR Repertoire After Bacillus Calmette–Guerin Treatment

Antigens released from tumor cell killing upon BCG treatment could expand and recruit effector and memory T cells leading to an overall increase in the diversity of TCR in the tissue microenvironment. TCR clonal proportion and clonal space homeostasis at pre and 3M post-BCG were then analyzed to further decipher the impact of BCG treatment on the diversity of TCR in the tumor microenvironment.

**Figure 4:**
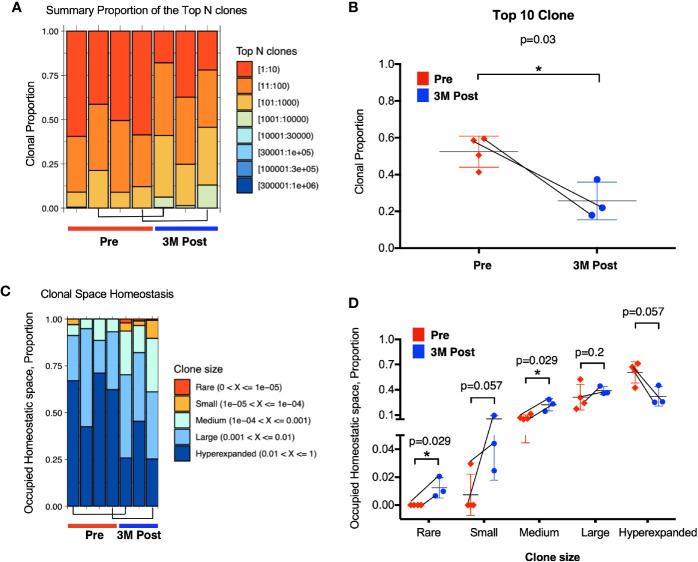
Enhanced TCR repertoire diversity after Bacillus Calmette-Guerin (BCG) treatment. **(A)** Clonal proportion of the top *n* clonotypes. Red bar represents the clonal proportion taken by the 10 most abundant clones. **(B)** Clonal proportion taken by top 10 most abundant clone at Pre (n=4, in red) and 3M post-BCG time point (n=3, in blue). 1 on Y-axis represents 100% of the total TCR repertoire. **(C)** Proportion of homeostasis space occupied by clonotypes classified as rare (0–0.00001), small (0.00001–0.0001), medium (0.0001–0.001), large (0.001–0.01), and hyperexpanded (0.01–1); 1 on Y-axis represents the 100% of the total TCR repertoire. **(D)** Proportion of occupied homeostatic space for pre (n=4, in red for each clone size) versus 3M post-BCG time points (n=3, in blue for each clone size) as classified by rare, small, medium, large, and hyperexpanded clone size, respectively. **(B, D)** Graphs show mean with standard deviation. *p<0.05 by Mann-Whitney U test. **(A–D)** Connecting lines showed two pairs of matched pre- and post-BCG samples.

Firstly, we analyzed the proportion of top 10 most abundant clonotypes of the total repertoire before and after BCG treatment. The top 10 most abundant clonotypes representing the majority of the repertoire was significantly lower in the post BCG tissues (mean = 0.2 or 20%) than the pre-BCG tissues (mean = 0.5 or 50%) (**Figures 4A, B**). This contraction of top 10 clonotypes, indicating that the repertoire has more newly expanded T cell clones potentially arising from BCG induced tumor reactive T cells after BCG treatment. To confirm this, we next analyzed the clonal space homeostasis by classifying clonotypes according to the TCR proportion taken up by clone size measured as rare (0–0.001%), small (0.001–0.01%), medium (0.01–0.1%), large (0.1–1%), or hyperexpanded (1-100%). In general, the post-BCG samples contained rarer, small or medium TCR clones, suggesting a higher diversity of TCR repertoire following BCG treatment (**Figure 4C**). Indeed, post-BCG T cell showed an expansion of clones classified as rare (respective mean of 0 versus 0.011, p=0.029), small (respective mean of 0.007 versus 0.055, p=0.057) and medium (respective mean of 0.073 versus 0.221, p=0.029) as compared to samples before treatment (**Figure 4D**). On the other hand, post-BCG T cell displayed contraction of clones classified as hyperexpanded (respective mean of 0.61 versus 0.32, p=0.057) (**Figure 4D**). In addition, we also examined the diversity indexes such as Shannon and Chao1 indices and indeed found a trend toward increased TCR diversity in post-BCG T cells (p= 0.057 for Chao1 index) (**Supplementary Figure S4A**). It should be noted that due to limited availability of tissue biopsies, the above comparisons were done using unmatched samples. However, we do observe a strong trend for the two matched samples (**Figures 4B, D**, and **Supplementary Figure S4A**) supporting our main claims above.

Taken together, the above data showed significant increase in smaller TCR clones suggesting the expansion of newly induced and potentially tumor-reactive T cells post-BCG.

### Baseline Densities of CD4+FOXP3- and CD8+PD-1+ T Cells Are Predictive of Response to Bacillus Calmette–Guerin Treatment

**Figure 5:**
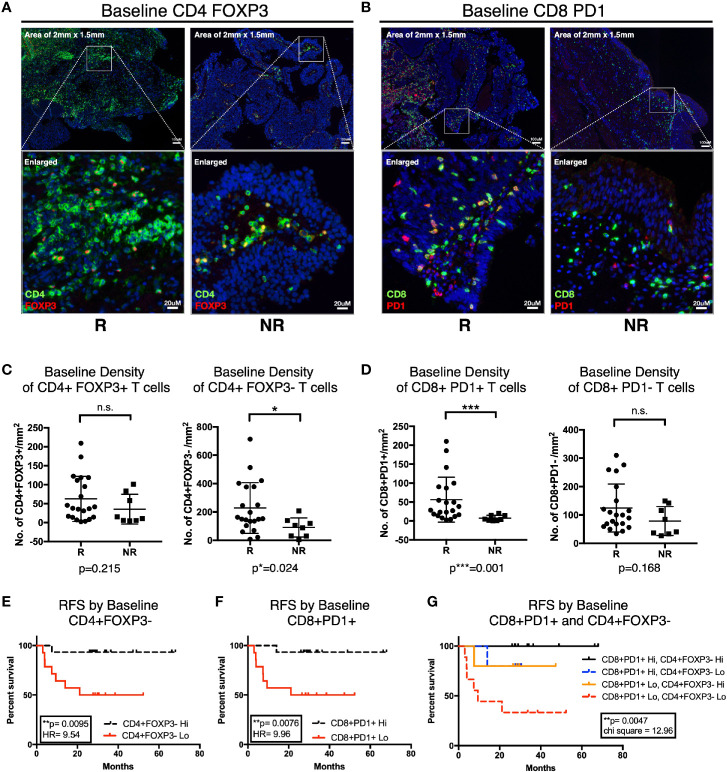
Higher baseline CD4^+^FOXP3^-^ and CD8^+^PD-1^+^ T cells is predictive of response to Bacillus Calmette-Guerin (BCG) treatment. **(A, B)** Representative multiplexed immunofluorescence image of tissue area (2 mm x 1.5 mm) with an enlarged image for Pre-BCG tissues, showing staining of **(A)** CD4 (green) and FOXP3 (red), **(B)** CD8 (green) and PD-1 (red), for both responders (R) and non-responders (NR), respectively. Scale bar equals to 100 um. **(C, D)** Quantification of baseline densities of **(C)** total CD4^+^FOXP3^+^, CD4^+^FOXP3^-^; **(D)** CD8^+^PD-1^+^, and CD8^+^PD-1^-^ T cells in R versus NR, respectively. ***p<0.001, *p<0.05 or not significant (n.s.) by two-tailed Mann-Whitney U test. **(E, F)** RFS for patients with low (red line) versus high (black line) baseline densities of **(E)** total CD4^+^FOXP3^-^ and **(F)** CD8^+^PD-1^+^ T cell. **(G)** RFS for patients with baseline densities of low CD8^+^PD-1^+^ and low CD4^+^FOXP3^-^ (red line); low CD8^+^PD-1^+^ and high CD4^+^FOXP3^-^ (orange line); high CD8^+^PD-1^+^ and low CD4^+^FOXP3^-^ (blue line); and high CD8^+^PD-1^+^ and high CD4^+^FOXP3^-^ (black line); respectively. **(E–G)** Low and high cell densities were < median versus > median. **p < 0.01 by Mantel-Cox log-rank test; HR, Hazard Ratio. RFS represents recurrence-free survival (months).

To further characterize the urothelial microenvironment before and after BCG treatment, we performed mIF staining on another larger independent FFPE validation cohort of 29 NMIBC patients (21 responders and eight non-responders; **Supplementary Table S2**) using multiplexed imaging platform as previously described (26). Given the upregulation of genes involved in T cell inhibitory function in post-BCG tissue transcriptome, we hence focused our mIF on CD4, FOXP3, CD8, and PD-1. We first examined the microenvironment of pre-BCG tumor tissues from 21 responders and 8 non-responders, in order to identify baseline biomarkers which could help us to predict for response to BCG treatment. We focused on the four major T cell subsets: CD4^+^FOXP3^+^ Treg, CD4^+^FOXP3^-^ non-Treg, CD8^+^PD-1^+^ and CD8^+^PD-1^-^ T cells (**Figures 5A, B**). We observed higher baseline densities of CD4^+^FOXP3^-^ non-Treg cells (p=0.024; **Figure 5C**) and CD8^+^PD-1^+^ T cells (p=0.001; **Figure 5D**) in responders versus non-responders. We then performed RFS analysis by log-rank (Mantel-Cox) test using the baseline densities of these two immune subsets. Indeed, we confirmed that patients who presented higher densities of baseline CD4^+^FOXP3^-^ T cells (Hazard ratio of 9.54, p=0.0095; **Figure 5E**) and CD8^+^PD-1^+^ T cells (Hazard ratio of 9.96, p=0.0076; **Figure 5F**) had a longer RFS compared to those who had lower baseline densities of these cells. This highlights that non-Treg, hence potentially active CD4^+^ T cells; and PD-1^+^, most likely pre-existing tumor reactive CD8 T cells, are important immune subsets contributing to subsequent response to BCG treatment. Of note, we confirmed that none of the baseline clinical characteristics such as gender, age, stage, grade, nor the BCG maintenance significantly impact on post-BCG recurrence free survival (RFS) by univariate analysis cox proportional hazards regression model (**Supplementary Table S7**).

More importantly, when we combined these two subsets in analysis, we observed that patients showing higher baseline densities of both CD8^+^PD-1^+^ and CD4^+^FOXP3^-^ T cell subsets had the most superior survival profile; whereas patients with low baseline densities of both of these cells had the worst recurrence-free survival profile (**Figure 5G**). Interestingly, comparing patients who had either high CD8^+^PD-1^+^ or high CD4^+^FOXP3^-^ alone, even though both showed improved RFS individually, we did not observe a vast difference in terms of their RFS profiles (**Figure 5G**). This indicates that both T cell subsets are important factors determining RFS after BCG treatment.

### Bacillus Calmette–Guerin-Induced Changes in Tissue Densities of CD4 and CD8 T Cells Is Linked to Response Status

**Figure 6:**
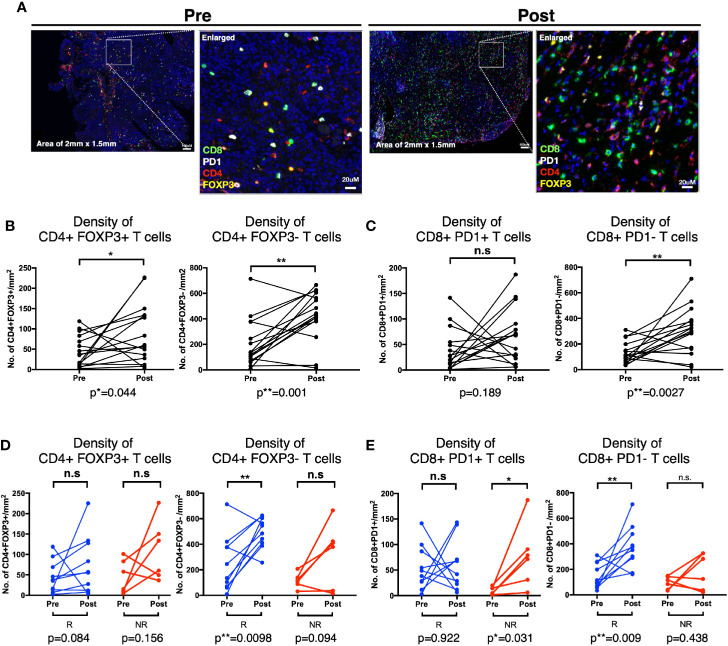
Distinct T cell subsets enriched in Post-Bacillus Calmette-Guerin (BCG) tissues according to response status. **(A)** Representative multiplexed immunofluorescence image of tissue area (2mm x 1.5mm) with an enlarged image for Pre- and Post-BCG tissues, with staining of CD8 (green), PD-1 (white), CD4 (red), and FOXP3 (yellow) shown. Scale bar equals to 100 um. **(B, C)** Quantification of densities of **(B)** total CD4^+^FOXP3^+^, CD4^+^FOXP3^-^; **(C)** CD8^+^PD-1^+^, and CD8^+^PD-1^-^ T cells, for Pre- and Post-BCG tissues. **(D, E)** Quantification of densities of **(D)** total CD4^+^FOXP3^+^, CD4^+^FOXP3^-^; **(E)** CD8^+^PD-1^+^, CD8^+^PD-1^-^ T cells, for Pre- and Post-BCG tissues, analyzed separately in Responders (R) versus Non-Responders (NR). Connected line specifies samples from same patient at different time points. **p < 0.01, *p < 0.05 or not significant (n.s.) by Wilcoxon matched-pairs signed rank test for pre and post analysis from the matched sample.

Lastly, to validate our findings from CyTOF and flow cytometry, that CD4 and CD8 T cells were reduced in peripheral blood post BCG treatment indicative of tissue recruitment, we then investigated the changes of the above mentioned four immune subsets comparing before and post BCG therapy, focusing on patients with available matched pre- and 3M post-BCG tissues (10 responders and 6 non-responders; **Supplementary Table S2**). Comparing pre and 3M post-BCG samples (**Figure 6A**), we observed significant higher densities of CD4^+^FOXP3^+^ Treg (p=0.044; **Figure 6B**), CD4^+^FOXP3^-^ non-Treg (p=0.001; **Figure 6B**) CD4 T cell subsets and CD8^+^PD-1^-^ T cells (p=0.0027; **Figure 6C**) in 3M post-BCG compared to pre-BCG. Whereas no difference was observed for CD8^+^PD-1^+^ T cells (median=68.2 cells/mm^2^ versus median=23.6 cells/mm^2^, p=0.189; **Figure 6C**). This shows a general increase in both CD4 and CD8 T cell subsets upon BCG induction.

The above data prompted us to further examine if these changes that are specifically linked to response status. Indeed, we found that the non-Treg CD4^+^FOXP3^-^ T cells were significantly increased after BCG therapy in responders (p=0.0098) but not in non-responders (p=0.094), whereas CD4^+^FOXP3^+^ Treg cells did not show any significant difference after BCG therapy in both responders (p=0.084) and non-responders (p=0.156) (**Figure 6D**). The post-treatment expansion of non-Treg cells indicates potential anti-tumor activity and response to BCG. Conversely, the non-exhausted or active CD8^+^PD-1^-^ T cells were significantly enriched in responders after BCG treatment (p=0.009) while CD8^+^PD-1^+^ T cells were significantly higher in non-responders (p=0.031) (**Figure 6E**). Interestingly, we found that the density of CD8^+^PD-1^+^ T cells were positively correlated with the PD-L1-expressing area in post- but not pre-BCG tumor tissues in non-responders (**Supplementary Figure S5**). This suggests that PD-1/PD-L1 exhaustion as a potential resistance pathway in patients non-responsive to BCG.

Taken together, we observed higher densities of non-Treg CD4^+^FOXP3^-^ T cells and active CD8^+^PD-1^-^ T cells in responders but a higher density of CD8^+^PD-1^+^ T cells in non-responders from post-BCG treated tissues. This data supports the rationale of combining anti-PD-1 treatment to overcome the resistance to BCG in NMIBC patients.

## Discussion

As the 5 years’ recurrence rate remains high for NMIBC patients treated with BCG, novel therapy design to improve BCG treatment is urgently needed. Understanding the mechanism especially the anti-tumor activity is key to unlock the hidden potential of BCG immunotherapy. In our current study, by combining high dimensional analyses, we uncovered immune activation upon BCG treatment in NMIBC patients which was reflected in the peripheral blood and local tissues. We identified a number of key immune subsets, particularly the CD4^+^ and CD8^+^ T cells where its reduction in frequency from the peripheral blood potentially indicated its tissue recruitment. From the transcriptomic analysis, flow cytometry assay and mIF on the tissues, we validated the tissues enrichment of key T cells subsets, upon BCG treatment. Importantly, we identified that baseline densities of non-Treg CD4^+^FOXP3^-^ and CD8^+^PD-1^+^ T cells as potential biomarkers to predict for response to BCG treatment in NMIBC patients. Finally, we found that response to BCG was associated with post-treatment increase of non-Treg CD4^+^FOXP3^-^ T cells and non-exhausted or active CD8^+^PD-1^-^ T cells in urothelial microenvironment; whereas failure of BCG treatment was related to higher densities of CD8^+^PD-1^+^ T cells in post-BCG tissues.

CD4 and CD8 T cells have been previously shown to play critical role in BCG-mediated anti tumor activity, mostly in animal models or inferred from markers related to T cell activities (7–11, 13). In our current study, we uncovered that the pre-BCG tumor tissues of responders were presented with higher densities of CD8^+^PD-1^+^ as well as non-Treg CD4^+^FOXP3^-^ T cells comparing with those of the non-responders (**Figures 5C, D**). More importantly, these high baseline densities of CD8^+^PD-1^+^ T cells and non-Treg CD4^+^FOXP3^-^ cells are associated with prolonged RFS upon BCG treatment (**Figures 5E, F**, **Supplementary Table S7**). Baseline Treg at cancer tissues prior to BCG treatment has been previously shown to correlate to BCG failure (9). However, our results of baseline FOXP3^+^CD4^+^ Treg cells did not show any significant difference in density between responders versus non-responders (**Figure 5C**). Instead, we observed a significant higher density of the non-Treg CD4^+^FOXP3^-^ cells at baseline (**Figure 5C**) that was further enhanced in post-BCG tissues from responders (**Figure 6D**), suggesting a more important role of the non-Treg cells in BCG response.

Previous evidence suggested that PD-1^+^CD8^+^ T cells could represent patient-specific tumor-reactive CD8 T cells infiltrating human tumors (37, 38). Remarkably, our present study showed that there were more baseline tissue CD8^+^PD-1^+^ T cells in responders compared to non-responders (**Figure 5D**), suggesting the presence of previously activated and potentially tumor reactive CD8 T cells in responders to BCG immunotherapy. In addition, densities of active CD8^+^PD-1^-^ T cells were increased in responders after BCG treatment (**Figure 6E**). We hypothesize that BCG treatment could reinvigorate the previously activated CD8 T cells which then leads to their expansion giving rise to new clonotypes. In this regard, we observed an increased clonal space occupied by rare clonotypes after BCG therapy (**Figures 4C, D**), inclining to support this hypothesis. Importantly, following the inflammation induced by BCG treatment, the CD8 T cells eventually became dysfunctional and the expression of co-inhibitory/exhaustion markers such as PD-1 were upregulated in tumor, especially in non-responders (**Figure 6E**). From our transcriptomic data, *CTLA4, TIM3* and *PD-L1* genes were enriched in post-BCG tissues (**Figure 3C**), indicating exhaustion state of post-BCG immune microenvironment.

As shown by our current findings, tumor escape with multiple coinhibitory or exhaustion molecules expressed by post-BCG tissues and infiltrating CD8 T cells, which could eventually lead to the resistance to BCG treatment. Here we described the increase of PD-1^+^ exhausted CD8 T cells post BCG therapy, were linked to non-responders of BCG treatment (**Figure 6E**). Therefore, it would be interesting to see if a sequential PD-1/PD-L1 blockade will improve the anti-tumor activity in BCG treatment, in these non-responding patients. Indeed, earlier this year, FDA approved Pembrolizumab (anti-PD-1) for treatment of patients with BCG-unresponsive, high risk NMIBC with CIS, who are ineligible for or have elected not to undergo cystectomy, based on its encouraging clinical trial result (KEYNOTE-057 study-NCT02625961) (39). Additionally, a number of ongoing clinical trials are currently investigating the efficacy of Pembrolizumab (NCT03711032) (40) and Nivolumab (another anti-PD-1 immunotherapy) (NCT03519256) (41) in neoadjuvant setting in combination of BCG, as compared to BCG alone, for patients with high risk NMIBC.

In conclusion, the current multidimensional immunoprofiling deciphered immune mechanisms upon BCG treatment in NMIBC patients. This study offers pre-treatment biomarkers capable of predicting response to BCG treatment and therefore preventing any delay in preventive treatment for high risk patients. It also supports the rationale of giving sequential PD-1/PD-L1 immunotherapy for high risk NMIBC patients who failed after adequate BCG therapy.

## Data Availability Statement

The original contributions presented in the study are publicly available. The RNA sequencing data can be found here: https://www.ebi.ac.uk/ega/studies/EGAS00001004764.

## Ethics Statement

The studies involving human participants were reviewed and approved by the Singapore General Hospital (SGH) institutional review board (IRB). The patients/participants provided their written informed consent to participate in this study.

## Author Contributions

SA, TC, and VC conceptualized and designed the study. TWC and VC supervised the study. CL, PN, YL, NN, CC, LL, SH, JL, and AL acquired the data. CL, PN, MW, PK, LK, JY, TC, and VC analyzed and interpreted the data. CL and VC wrote the manuscript. LL, LK, JY, and TL provided technical and material support. All authors contributed to the article and approved the submitted version.

## Funding

This study was supported by the National Medical Research Council (NMRC), Singapore (reference numbers: TA/0005/2012, TCR15Jun006, CIRG16may048, CSAS16Nov006, CSASI17may003, and LCG17MAY003).

## Conflict of Interest

The authors declare that the research was conducted in the absence of any commercial or financial relationships that could be construed as a potential conflict of interest.
