# Supplementary material for: Immunological Hallmarks for Clinical Response to BCG in Bladder Cancer
Source: Front Immunol. 2021 Jan 29;11:615091. doi: 10.3389/fimmu.2020.615091 (PMC7879685; doi:10.3389/fimmu.2020.615091)
Supplement: Supplementary file 1 [file DataSheet_1.pdf]

Supplementary Material

Supplementary Table S1: Clinical and demographic information of five NMIBC patients.

Pat ID	Age	Gender	Race	Stage (TNM)	Grade	Tumour size (mm)	Response to BCG
BCGX02	72	Male	Chinese	T1	HG ,CIS	23x19x3	R
BCGX09	67	Male	Chinese	T1	HG	12x7x3	R
BCGX10	74	Male	Chinese	Ta	HG	Multiple; max-5	R
BCGX15	56	Male	Indian	T1	HG	12x10x3	R
BCGX16	68	Male	Chinese	T1	HG	20x5x2	R

Footnote:
Stage TNM: 2009 TNM system
HG: High Grade
CIS: Carcinoma In-situ
R: Responder

Supplementary Table S2. Clinical information of the 29 NMIBC patients (FFPE cohort) for multiplexed immunofluorescence by VECTRA.

Characteristics	All patients (n=29)	Responders (n=21)	Non-responders (n=8)
Gender			
Male (%)	21 (72.4%)	14 (66.7%)	7 (87.5%)
Female (%)	8 (27.6%)	7 (33.3%)	1 (12.5%)
Age#, years (range)	71 (56-86)	70 (56-84)	76 (64-86)
Stage			
Ta (%)	11 (37.9%)	9 (42.9%)	2 (12.5%)
T1 (%)	16 (55.2%)	10 (47.6%)	6 (87.5%)
CIS (%)	2 (6.9%)	2 (9.5%)	0
CIS associated ^ (%)	7 (24.1%)	6 (28.6%)	1 (12.5%)
Grade			
Low grade (%)	2 (6.9%)	2 (9.5%)	0
High grade (%)	27 (69.0%)	19 (61.9%)	8 (87.5%)
At least one BCG maintenance administered (%)	14 (48.3%)	11 (52.4%)	3 (37.5%)
Follow up period from BCG therapy#, months (range)	N.A.	33.5 (26.0-81.0)	N.A.
Time to Tumour Recurrence#, months (range)	N.A.	N.A.	7.4 (3.0-21.1)

Footnote:
Stage TNM: 2009 TNM system
CIS: Carcinoma In-situ
median is shown
^ Any stage disease associated with CIS or Stage=CIS

Supplementary Table S3. Antibodies used for CyTOF staining

Lymphoid panel

Isotopes	Antibodies	Clone	Vendor
89	CD45 (Barcode 1)	HI30	Fluidigm
112/114	CD14	TüK4	Lifetechnologies
115	CD45 (Barcode 2)	HI30	Biolegend
139	CD3	UCHT1	Biolegend
141	CD19	HIB19	Biolegend
142	CD45RO	UCHL1	Biolegend
143	HLA-DR	L243	Biolegend
144	CD8	SK1	Biolegend
145	T-bet	4B10	Biolegend
146	γδTCR	B1	Biolegend
147	PD-1	EH12.2H7	Biolegend
148	CD4	SK3	Biolegend
149	iNKT	6B11	Biolegend
150	CD103	B-Ly7	Ebioscience
151	GATA3	TWAJ	Ebioscience
152	TNFα	Mab11	Biolegend
153	CD25	2A3	BD bioscience
154	CD27	O323	Biolegend
155	CD152	BNI3	BD bioscience
156	PD-L1	29E.2A3	Biolegend
157	CD244	C1.7	Biolegend
158	IL-10	JES3-9D7	Biolegend
159	LAG-3	17B4	Abcam
160	TIM-3	F38-2E2	Biolegend
161	CCR7	G043H7	Biolegend
162	CD56	NCAM16.2	BD bioscience
163	CXCR3	G025H7	Biolegend
164	CD161	HP-3G10	Biolegend
165	FoxP3	PCH101	Ebioscience
166	Ki67	20Raj1	Ebioscience
167	CD80	2D10	Biolegend
168	IFN-γ	B27	Biolegend
169	IL-17A	BL168	Biolegend
170	EOMES	21Mags8	Ebioscience
171	CD45RA	JS-83	Ebioscience
172	CD45 (Barcode 3)	HI30	Biolegend
173	GranzymeB	CLB-GB11	Abcam
174	CD137	4B4-1	Biolegend
175	CCR5	T21/8	Biolegend
176	CD69	FN50	Biolegend
191/193	Ir Intercalator		Fluidigm

Myeloid panel

Isotopes	Antibodies	Clone	Vendor
89	CD45 (Barcode 1)	HI30	Fluidigm
112/114	CD14	TüK4	Lifetechnologies
115	CD45 (Barcode 2)	HI30	Biolegend
139	HLA-DR	L243	Biolegend
143	CD3	UCHT1	Biolegend
151	CD1c	L161	Biolegend
154	CD15	H198	Biolegend
159	CD11c	Bu15	Biolegend
161	CD56	NCAM16.2	BD bioscience
167	CD19	HIB19	Biolegend
172	CD45 (Barcode 3)	HI30	Biolegend
174	CD33	WM53	Biolegend
191/193	Ir Intercalator		Fluidigm

Supplementary Table S4. List of antibodies used in Flow Cytometry

Flurochrome	Antibodies	Clone	Vendor
APC/Cy7	CD45	HI30	Biolegend
Brilliant Violet 605	CD4	OKT4	Biolegend
PerCP/Cy5.5	CD8	RPA-T8	Biolegend
Alexa Fluor 700	CD3	UCHT1	Biolegend
Pacific Blue	CD16	3G8	Biolegend
FITC	γδTCR	B1	Biolegend
PE-CF594	CD56	NCAM16.2	BD Bioscience
PE/Cy7	CD69	FN50	Biolegend
APC	PD-1	EH12.2H7	Biolegend
Brilliant Violet 510	CD27	M-T271	Biolegend
DAPI	Live/Dead	-	BD Bioscience

Supplementary Table S5. Phenotype of four FlowSOM clusters and its p-value by repeated measure ANOVA across three time points.

FlowSOM Cluster	Phenotype	p-value (RMA)
**7**	CD3+CD4+CD25+FOXP3+	0.017
**13**	CD3+CD4+CD45RO+Tbet+	0.025
**24**	CD56+CD3+GB+	0.066
**36**	CD3+CD8+TIM3+PD-1-GB+	0.086

Supplementary Table S6. Pathways enriched in either pre-BCG or post-BCG tissues according to DAVID pathway enrichment analysis on DEGs.

Enriched pathways	Benjamini	Genes enriched in PreBCG
IPR000884:Thrombospondin, type 1 repeat	3.8446E-04	SEMA5A, UNC5B, ADAMTS13, ADAMTSL3, C6, RSPO2, THBS2, ADAMTS5, SPON1, ADGRB2
Ion transport	1.1775E-02	TRPC4, SLC9A4, KCNB2, GRIK3, SLC9A2, ANO1, CLDN10, ATP1A2, KCNK2, KCNIP4, KCNS3, P2RX6, GRIA1, RYR3, KCNN2, SLC22A3, SCN4B, SLC30A2, SCNN1G, SLC5A7, SCNN1B, SLC5A12
Insulin-like growth factor binding protein, N-terminal	4.5008E-02	EPHA5, MATN2, ERBB4, FBLN2, SCUBE2, CRTAC1, KAZALD1, RSPO2, PCSK6, SLIT3

Enriched pathways	Benjamini	Genes enriched in PostBCG
GO:0031295~T cell costimulation	7.8892E-13	HLA-DQB2, CD3G, CD3D, CD3E, CD247, CTLA4, CCL19, HLA-DQA1, PDCD1LG2, BTLA, CD86, TNFSF13B, CCL21, CD40LG, ICOS, CD274, GRAP2, CD5, CD28
GO:0070098~chemokine-mediated signaling pathway	3.5111E-11	CXCL1, CCL1, CMKLR1, CXCL9, CXCR1, CXCL8, CCL19, CCL4L2, CXCL6, CXCR3, CCL4, CCL18, CCL17, CCL22, CCL20, CXCR4, CCL21
GO:0006935~chemotaxis	1.3563E-09	CCL1, CXCL1, CMKLR1, CXCL9, FPR1, CXCR1, CXCL8, NCKAP1L, CXCL6, CXCR3, CCL18, LGALS9, CCL17, PLAUR, CCL22, CCR7, CCL20, CXCR4, PTAFR
GO:0071347~cellular response to interleukin-1	4.9117E-07	CCL1, CHI3L1, CXCL8, CCL19, CCL4L2, CCL4, CCL18, CCL17, LCN2, CCL22, CCL20, CCL21, PYCARD
GO:0070374~positive regulation of ERK1 and ERK2 cascade	1.8718E-06	HAVCR2, CCL1, CHI3L1, PTPN22, CCL19, CCL4L2, CCL4, SLAMF1, LGALS9, CCL18, CCL17, CCL22, CCR7, CCL20, CCL21, RASGRP1, PYCARD, TREM2
GO:0071346~cellular response to interferon-gamma	5.5186E-06	CCL1, CCL22, GBP5, CCL20, CCL21, CCL19, CCL4L2, CCL4, CCL18, LGALS9, CCL17
GO:0071356~cellular response to tumor necrosis factor	7.5322E-06	CCL1, CHI3L1, CXCL8, CCL19, CCL4L2, CCL4, CCL18, CCL17, LCN2, CCL22, IL18BP, CCL20, CCL21, PYCARD
GO:0050852~T cell receptor signaling pathway	7.3554E-04	HLA-DQB2, CD3G, CD3D, CD3E, CD247, PTPN22, FOXP3, TRAT1, HLA-DQA1, GRAP2, THEMIS2, CD28, LCP2
GO:0002407~dendritic cell chemotaxis	6.8938E-03	CCR7, CXCR4, CCL21, CXCR1, CCL19
GO:0050707~regulation of cytokine secretion	2.1836E-02	TLR10, TLR1, TLR2, TLR8

Supplementary Table S7. Univariate analysis for RFS by baseline charateristics of the 29 NMIBC patients (FFPE cohort).

	Univariate Analysis by Cox Proportional Hazards Regression Model		
**Baseline Characteristics**	**Hazard Ratio**	**Hazard Ratio 95% CI**	**p-value**
Gender (0:Female ; 1:Male)	2.93	0.36-23.83	0.31
Age^ (median = 71 years)	2.44	0.49-12.12	0.27
Stage (0:pTa ; 1:pT1 ; 2:CIS)	1.19	0.39-3.63	0.76
Grade (0:Low grade ; 1:High grade)	0	0-Infinity	1
CIS associated[#]	0.37	0.05-3.03	0.36
BCG Maintenance administered[$]	0.79	0.20-3.15	0.73
CD4+ counts^	0.10	0.01-0.85	***0.034****
CD8+ counts^	0.45	0.11-1.89	0.27
CD4+FOXP3+ counts^	0.55	0.13-2.32	0.42
CD4+FOXP3- counts^	0.10	0.01-0.85	***0.034****
CD8+PD1+ counts^	0.10	0.01-0.81	***0.031****
CD8+PD-1- counts^	0.45	0.11-1.89	0.27
CD8+PD-1+ and CD4+FOXP3- counts[Δ]	0.15	0.04-0.59	***0.007*****

Footnote:
RFS: recurrence free survival
CIS: Carcinoma In-situ
^ < versus ≥ median
# CIS associated: 0:None ; 1:Any stage disease associated with CIS or Stage=CIS
$ BCG Maintenance administered: 0:None ; 1:At least one maintenance given post BCG induction before Relapse or Last follow up
Δ 1: CD8+PD-1+ Low (< median) CD4+FOXP3- Low (< median)
2: CD8+PD-1+ Low (< median) CD4+FOXP3- High (≥ median) or CD8+PD-1+ High (≥ median) CD4+FOXP3- Low (< median)
3. CD8+PD-1+ High (≥ median) CD4+FOXP3- High (≥ median)

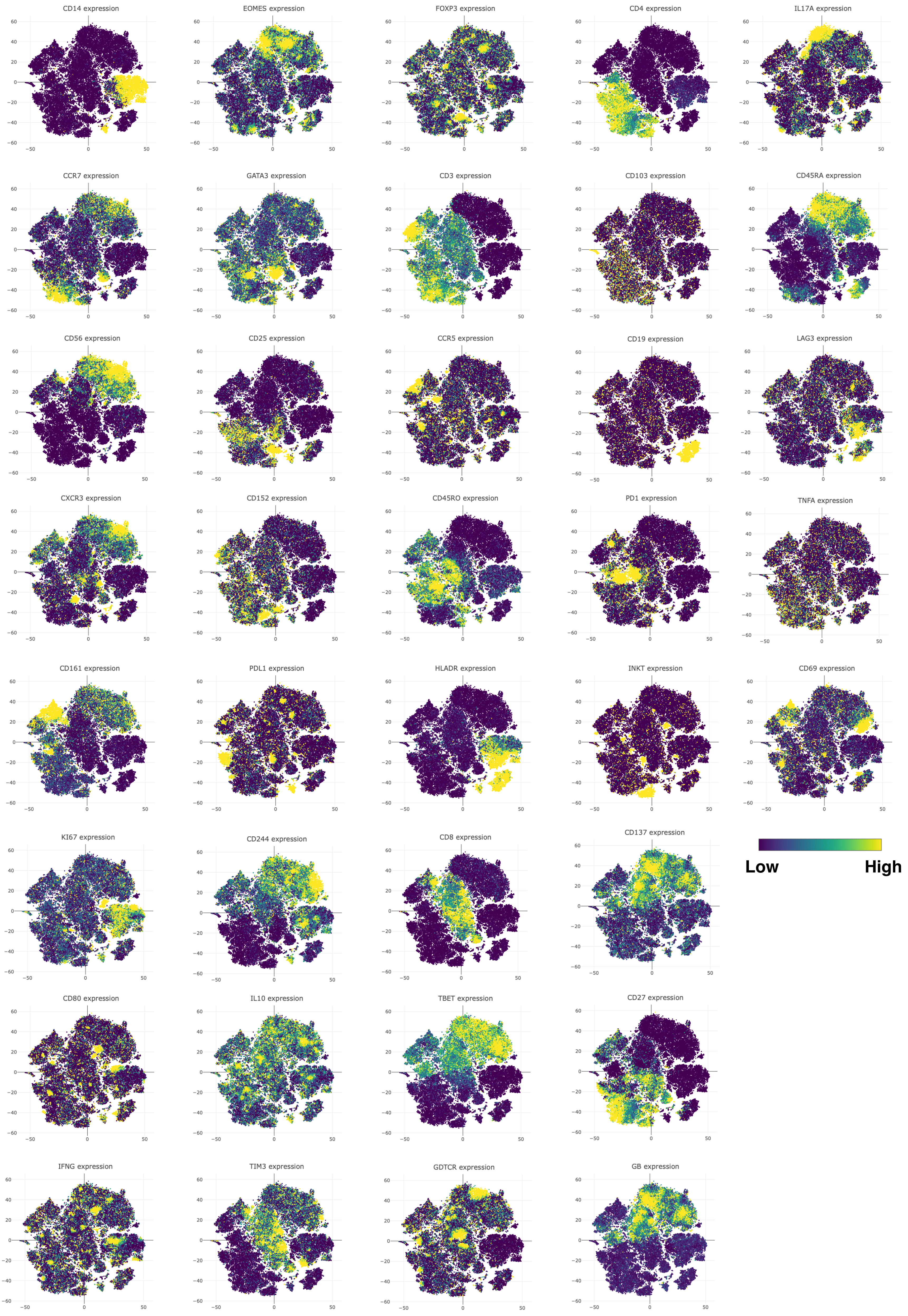

Supplementary Figure S1. t-SNE plots showing relative expression of 37 markers individually. Each dot represents a single cell and cells are coloured according to relative expression of the indicated marker. Axes show arbitrary units based on t-SNE algorithm.

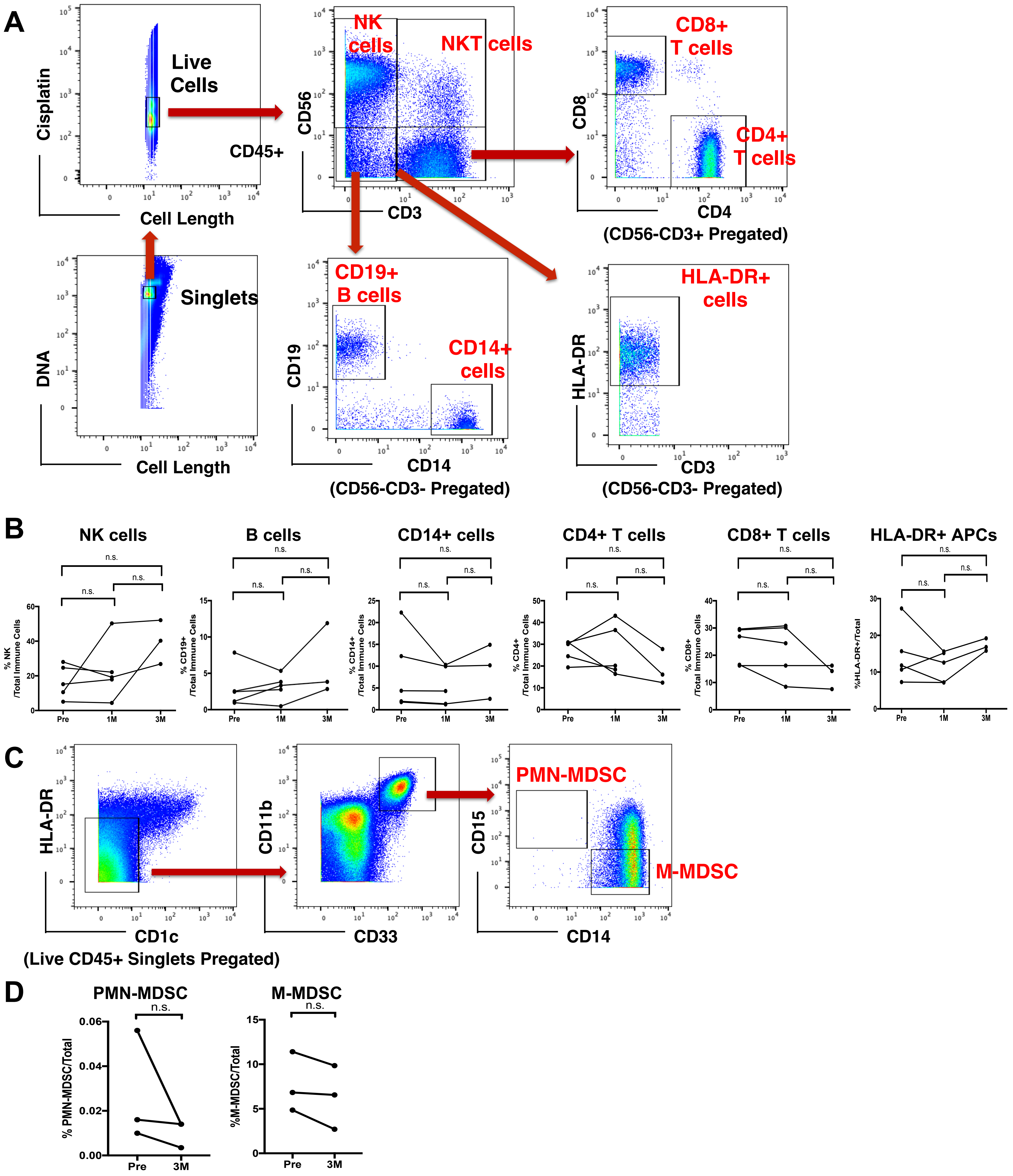

Supplementary Figure S2. Gating strategies. **A,**Representative dot plots showing six immune lineages and HLA-DR+ cells gated using FlowJo. Singlets were gated out as cells positive for iridium DNA intercalator (DNA+, y-axis) of a specific cell length (x-axis). All live immune cells were gated for Cisplatin(Live/Dead dye)- CD45+ populations. The six immune lineages were gated using their respective immune markers: NK cells (CD56+CD3-); NKT cells (CD56+CD3+); CD19+ B cells (CD56-CD3-CD19+); CD14+ cells (CD56-CD3-CD14+); CD4+ T cells (CD56-CD3+CD4+); CD8+ T cells (CD56-CD3+CD8+) and HLA-DR+ cells. **B,** Frequencies of NK cells, CD19+ cells, CD14+ cells, CD4+ T cells, CD8+ T cells and HLA-DR+ antigen-presenting cells (APCs); from all PBMC samples at Pre, 1 months Post BCG and 3 months Post BCG time points. Connected line specifies samples from same patient at different time points. Not significant (n.s.) by multiple pairwise comparison using Dunn's test for data points with complete set of three time points from three patients. **C,** Representative dot plots showing two main myeloid-derived suppresor cells (MDSC) gated using FlowJo: polymorphonuclear-MDSC (PMN-MDSC) and monocytic-MDSC (M-MDSC). **D,** Frequencies of PMN-MDSC and M-MDSC of total live immune cells from PBMCs at Pre- and 3 months Post BCG time points. Not significant (n.s.) by Wilcoxon matched-pairs signed rank test for pre and post analysis with the matched sample.

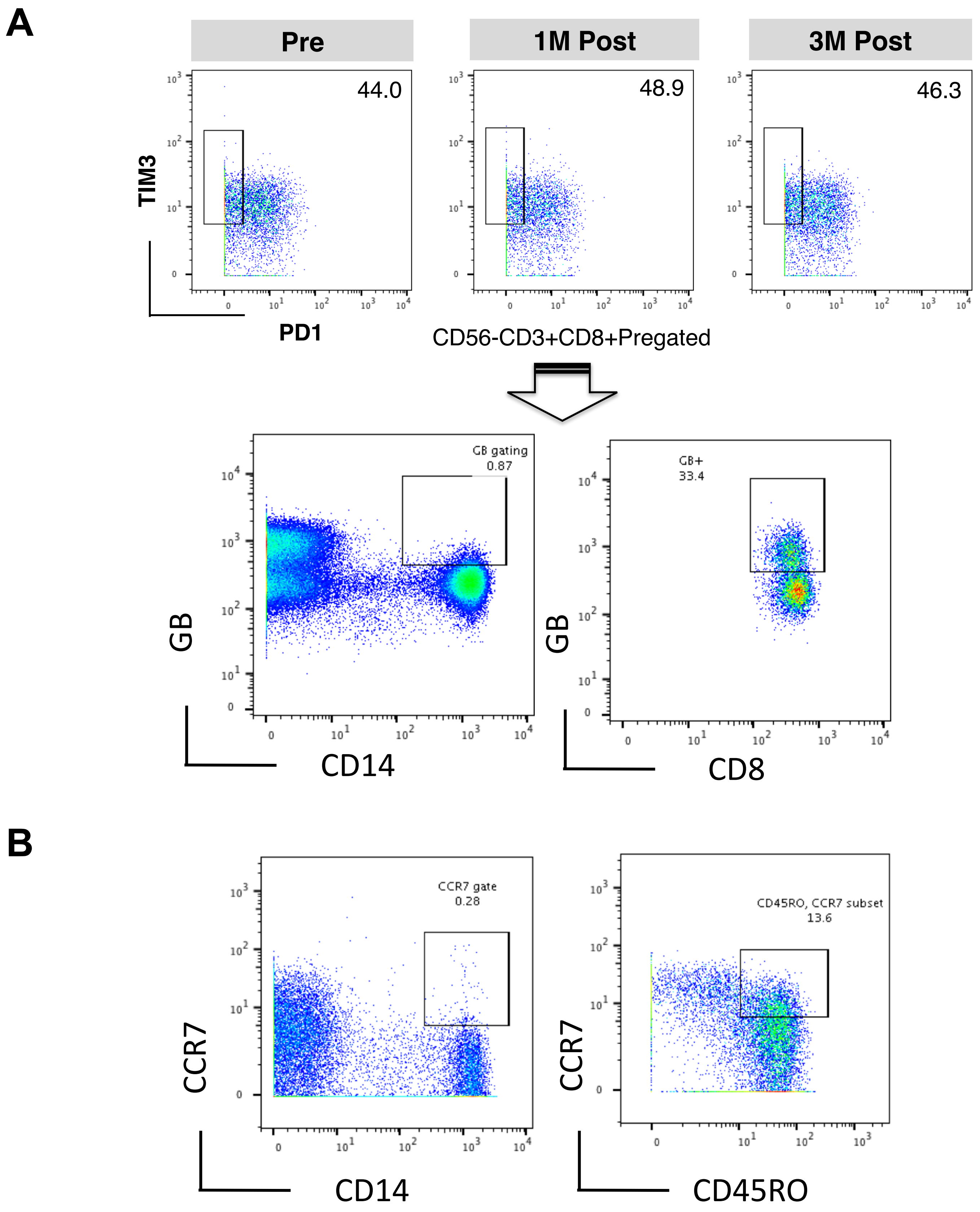

Supplementary Figure S3. Gating strategies for cluster 36 and 13.

A, Top, Representative dot plots showing TIM3+PD1- cells for pregated CD56-CD3+CD8+ from patient 16 at three time points. Bottom, representative dot plots showing gating for granzyme B (GB) based on CD14+ subset which do not express GB versus CD8+ T cells that express GB**. B,** Representative dot plots from patient 16 showing gating for CCR7 based on CD14+ subset which do not express CCR7 versus CD4+ T cells that express CCR7.

A

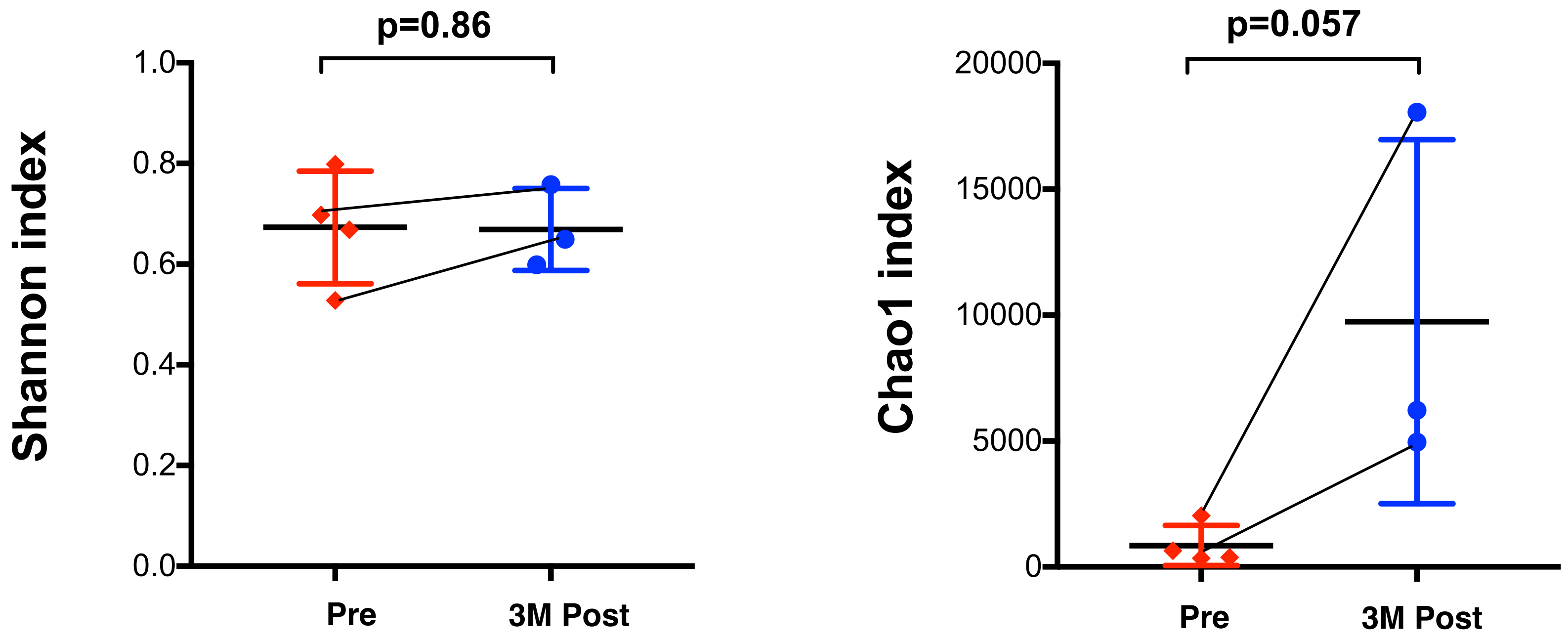

Supplementary Figure S4. Scatter dot plot representation of diversity indices (Shannon) and richness indices (Chao1) at Pre and 3M Post-BCG time point. A, Graph shows Shannon indices (left) and Chao1 indices (right) of pre- and post-BCG samples. P values by Mann-Whitney U test. Connecting lines showed samples with matched pre- and post-BCG samples.

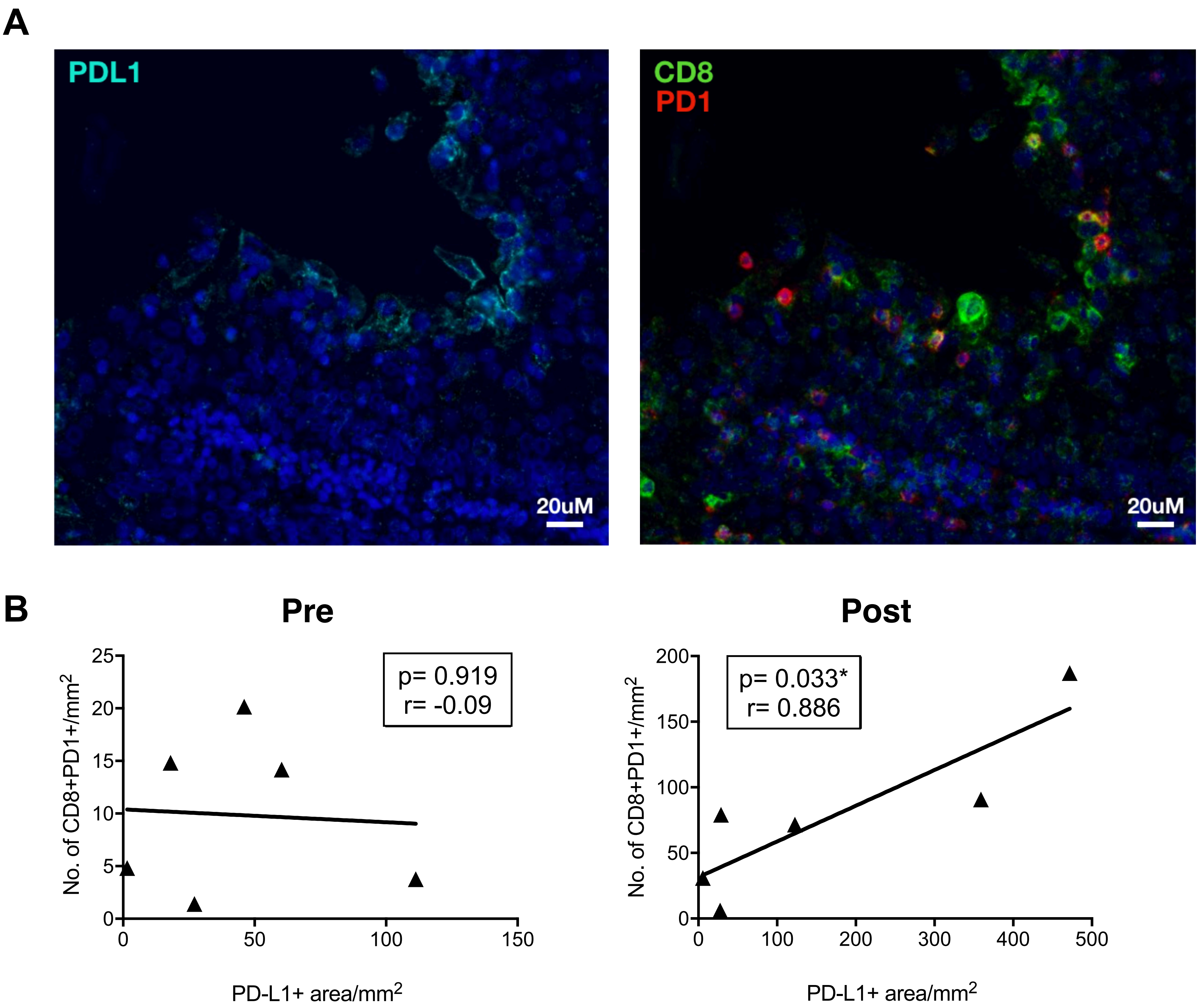

Supplementary Figure S5. Correlation of PD-L1 with CD8+PD1+ cells in Pre- and Post-BCG treated tumor tissues in Non-Responders. A, Left panel: Representative immunofluorescence image of individual staining of PD-L1 (cyan) on consecutive tissue section. **Right panel:** Representative multiplexed immunofluorescence image showing CD8 (green), PD-1(red) and DAPI (blue). Scale bar equals to 20µm. **B,** Correlation of density of PD-L1+ area with CD8+PD-1+ cells in Pre- (left) and Post- (right) treated tumor tissues in Non-Responders. PD-L1+ area was quantified as pixel using ImageJ software, including staining in both tumor cells and tumor-infiltrating immune cells; p values by Spearman correlation test. * $p < 0.05$. r= spearman's coefficient, rho.
